# A Rare Cervical Dystonia Mimic in Adults: Congenital Muscular Torticollis (Fibromatosis colli), a Follow-up

**DOI:** 10.3389/fneur.2016.00007

**Published:** 2016-02-01

**Authors:** Mehmet C. Uluer, Branko Bojovic

**Affiliations:** ^1^Department of Surgery, University of Maryland School of Medicine, Baltimore, MD, USA; ^2^Division of Plastic, Reconstructive and Maxillofacial Surgery, R Adams Cowley Shock Trauma Center, University of Maryland Medical Center, Baltimore, MD, USA

**Keywords:** fibromatosis colli, congenital muscular torticollis, muscular torticollis, sternocleidomastoid muscle, surgical procedures, operative

## Abstract

Neglected or undiagnosed congenital muscular torticollis in adults is quite rare, although it is the third most common congenital deformity in the newborn (1). When left untreated at an early age, deficits in lateral and rotational range of motion can occur along with irreversible facial and skeletal deformities that develop over time. Subtle cases can go unnoticed until early adulthood, with predominant fibrotic replacement in the sternocleidomastoid (SCM) making physical therapy and chemodenervation mostly ineffective. Surgical intervention, in these cases, can prove effective in alleviating pain, improving function and cosmesis (2). We report an update on a previously reported case, misdiagnosed as cervical dystonia, which had undergone partial myectomy of the anterior belly of the SCM with some relief of symptoms but without total resolution after the correct diagnosis of fibromatosis colli (3).

## Case Report

**Figure 1:**
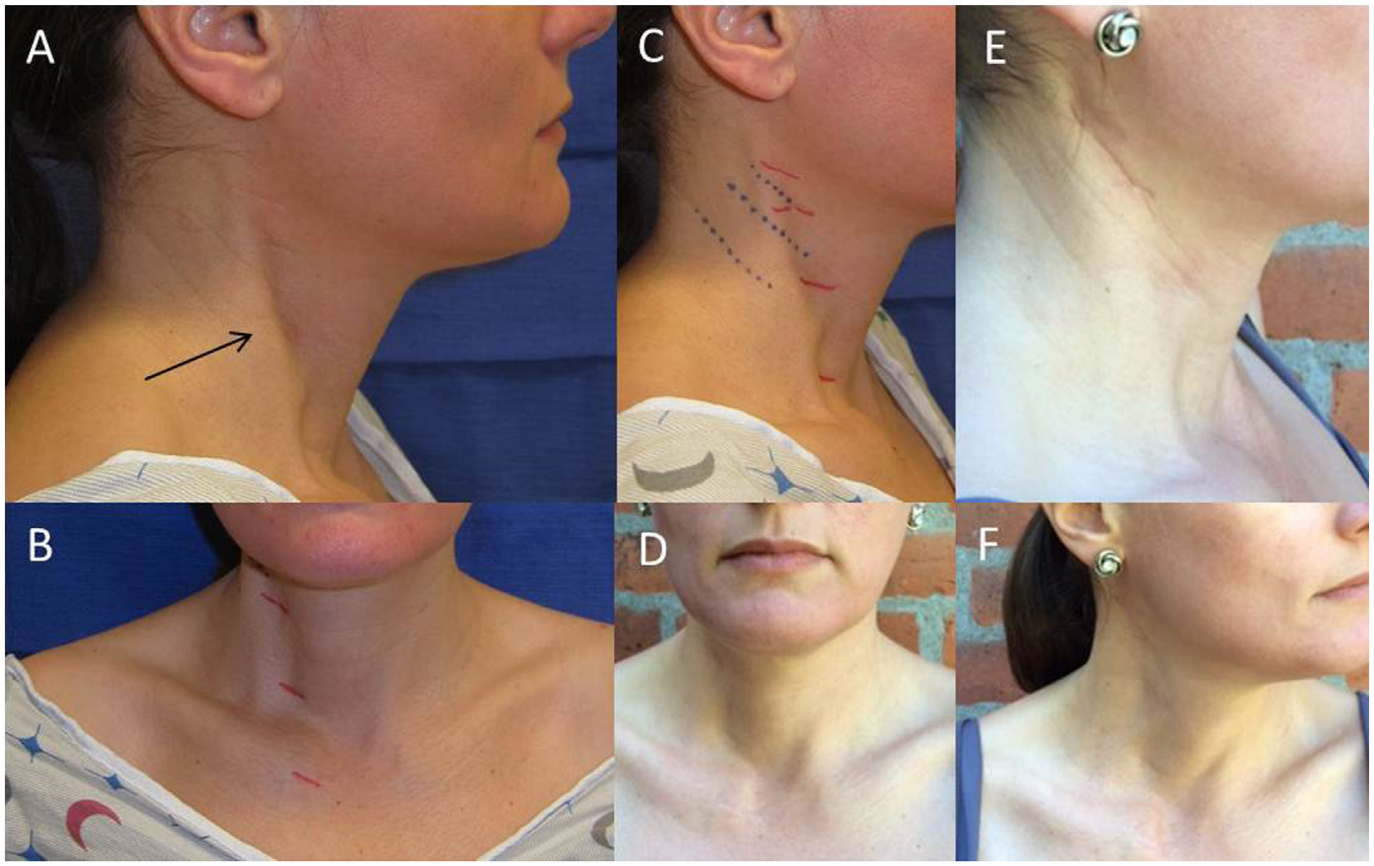
**Images of tethering and anterior SCM defect pre-operatively and post-operative resolution of marked tethering and appearance of incision**. Noticeable tethering from the lateral view marked with an arrow **(A)** and from the anterior view with previous stair-step incision marked in red **(B)**. Lateral view of pre-operative planning for Z-plasty incision with Langer lines indicated in blue **(C)**. Two views of post-operative decrease in tethering **(D,F)**. Post-operative appearance of Z-plasty incision at 6 months **(E)**.

**Figure 2:**
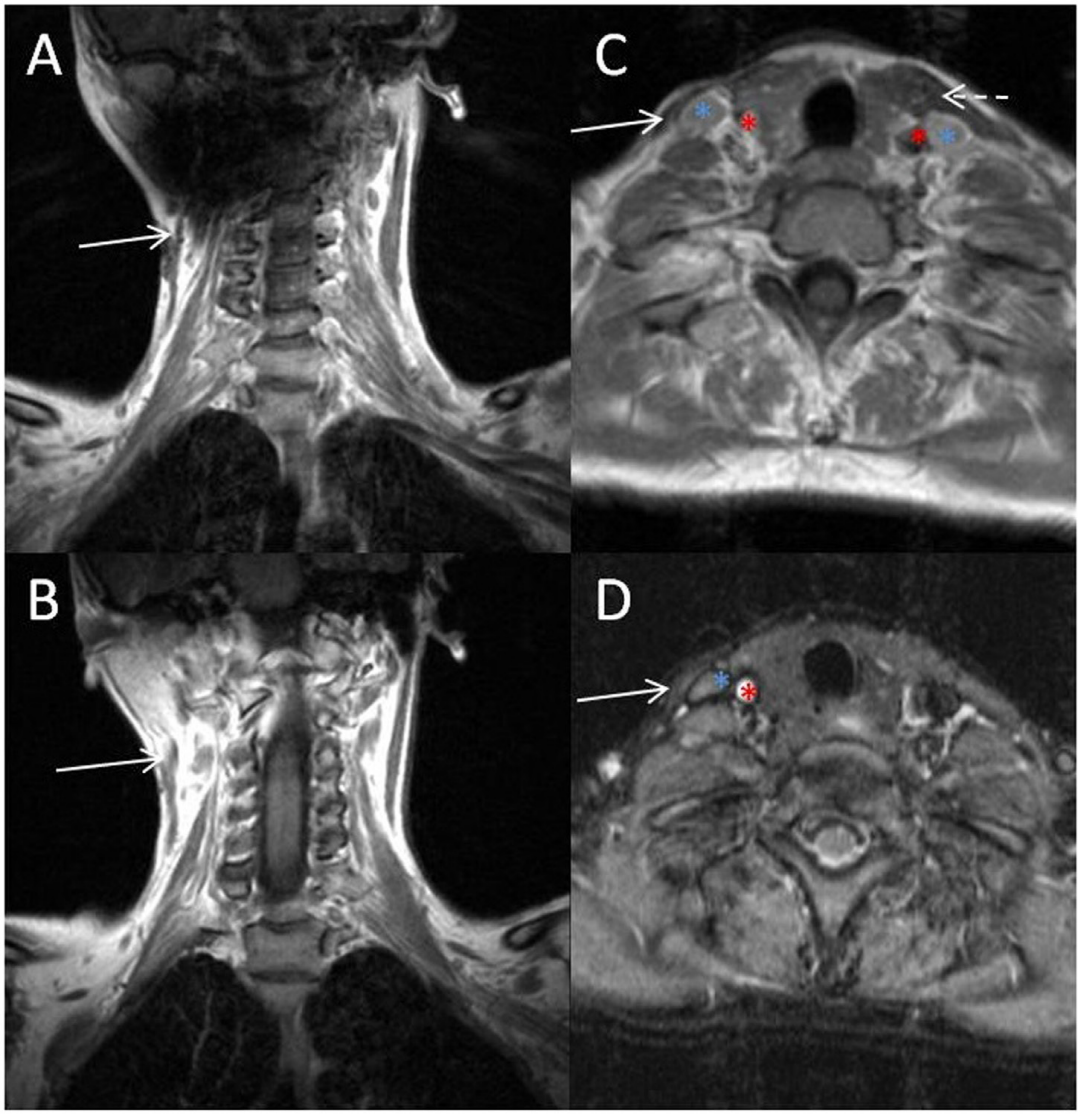
**MRI of fibrotic band and remnant SCM**. Sequential coronal **(A,B)** and axial **(C,D)** images of the fibrotic band (solid arrow) on coronal sequences, remnant SCM (solid arrow) on axial images and the carotid artery (red asterisk), internal jugular vein (blue asterisk), and left SCM (dashed arrow) are indicated for reference.

**Figure 3:**
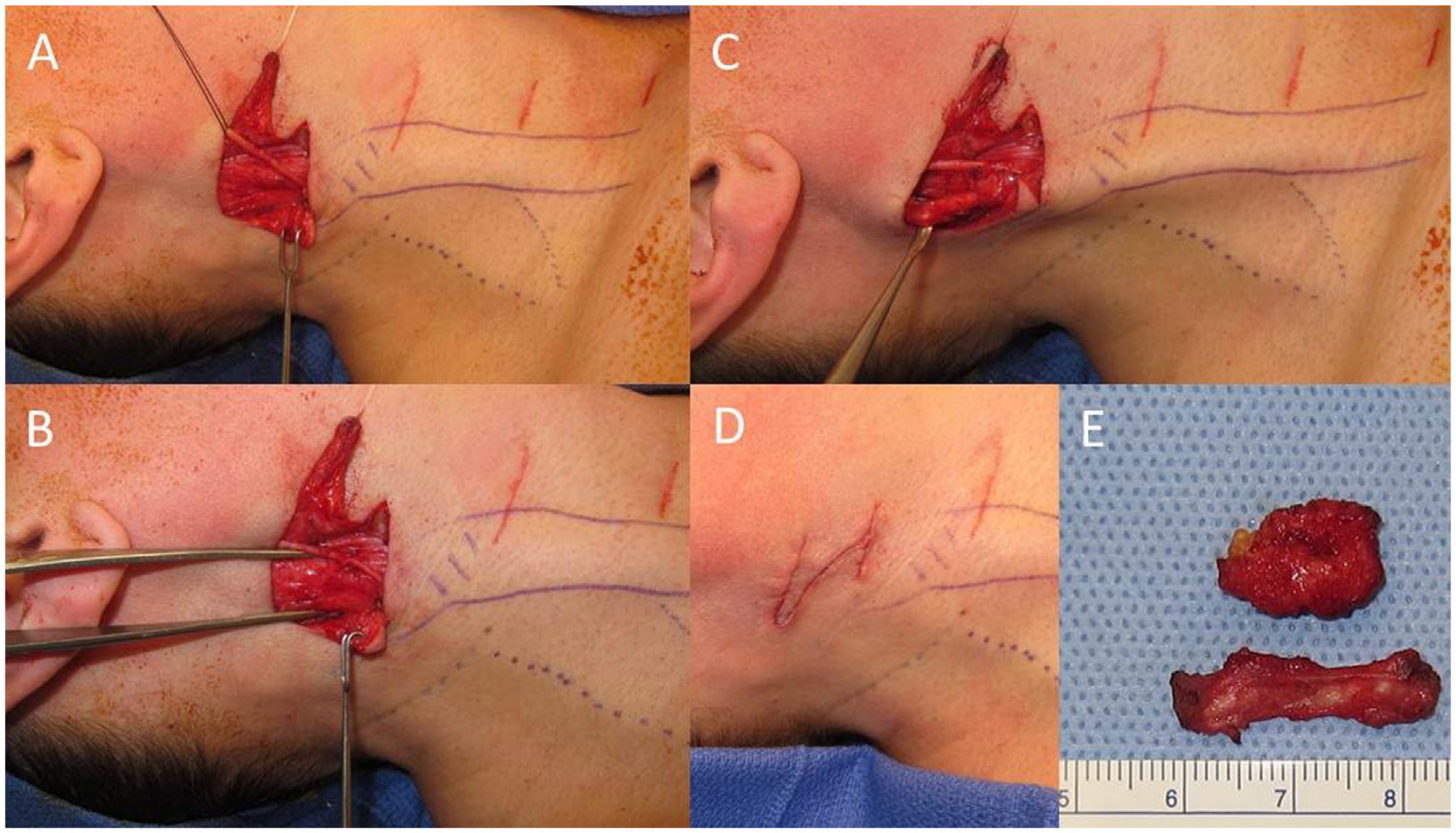
**Intraoperative images and fibrotic band**. Initial dissection with isolation and neurolysis of the great auricular nerve indicated by the black suture **(A)**. Right sternocleidomastoid (SCM) indicated by the forceps **(B)**. Fibrotic band within the SCM **(C)**. Closure of Z-plasty incision **(D)** after resection of fibrotic band and remnant SCM **(E)**.

A 42-year old woman, who noticed right head tilt and bilateral shoulder pain in her late twenties, had previously undergone resection of the anterior belly of the right sternocleidomastoid (SCM) 5 years ago after failed physical therapy and Botox injections. Her initial operation was performed in a stair-step fashion, with five transverse incisions from the insertion of the SCM on the mastoid to the origin at the sternum. She had resolution of torticollis and was able to maintain appropriate midline posture after this procedure, though continued to report pain (up to 6/10 at its worst on a visual analog scale), tightness, limited range of motion, and postural worsening. Pre-operatively she did not have any craniofacial asymmetry. Pathology from the previous operation showed fibroadipose tissue and skeletal muscle showing fibrosis, pathognomonic for congenital muscular torticollis (CMT). On physical exam, a range of motion deficit and weakness was not evident. Blunting and diminution of the sternal head was seen with a still intact mastoid and clavicular head. Additionally on physical exam, a localized band of tissue with tethering could be palpated within the superior portion of the remaining SCM with rotation to the left, a taut right SCM can be seen along the entire remnant of the muscle (Figure 1). MRI prior to the second operative intervention showed marked atrophy of the right SCM and a persistent fibrotic band just deep to the skin (Figure 2). Given these findings and subsequent discussions with the patient, a second surgical intervention was decided to alleviate her symptoms. This was done by performing a Z-plasty incision joining the previous two superior incisions, followed by neurolysis of the great auricular nerve and circumferential release of the area causing the tethering (Figure 3). Neurolysis of the great auricular nerve was done due to the extensive scaring in the area, preventing mobilization of the remnant SCM and presenting a high risk for injury. Bipolar electrocautery and nerve stimulator were used to avoid damaging the spinal accessory nerve throughout the surgery. Due to the ineffectiveness of the previous bipolar release of only the anterior belly at the origin and insertion, and the possibility of re-scarring in the absence of supervised, consistent physical therapy after her first operation a complete focused unipolar release at the insertion of the posterior belly with resection of the observed band and partial resection of the remnant posterior belly was opted due to patient preference for cosmesis. Discussions with the patient clarified that intraoperative investigation with unsatisfactory results might require a release inferiorly if results were not obtained by this approach. During the operation, a 3.5-cm section of fibrotic tissue was completely resected along with the remnant posterior belly of the SCM, revealing benign fibroadipose tissue, fibrotic skeletal muscle, and nerve with focal atrophic and reactive changes (Figure 3B). Complete resection of this band and the remaining portion of the posterior SCM at its insertion to the mastoid resulted in near complete alleviation of her symptoms per the patient; she in fact felt improvement in the recovery room. Six months out from surgery, with 2 months of post-operative extensive physical therapy and continued daily exercises, she now reports pain of 1/10 at its worst. She also reports resolution of any unpleasant sensation in the area of her previous incisions which might be due to release and decompression of the great auricular nerve. Overall, the patient states that she has had an 80–85% improvement in the pain, tightness, limited range of motion, and postural worsening experienced prior to surgery and a 90–95% improvement with regards to her pre-operative pain, which was her primary source of discomfort.

## Discussion

Surgical treatment of neglected CMT in adults can be difficult due to adjacent tissue contractures, adhesions, and extensive fibrosis (4, 5). In this case, the initial operation done by releasing the anterior belly of the SCM at the origin and insertion (bipolar release of only one muscle belly) by accessing it along its whole length through a stair-step fashion was not completely effective. It is possible portions of the fibrotic band were missed with the initial partial myectomy or re-scarring might have occurred. In the literature, there are very few examples of reoperation to release residual bands (6, 7), most patients in presented case series do not require or have not undergone any further intervention after the initial operation (2, 8, 9). Additionally, most of these cases have undergone bipolar release or selective unipolar release involving both muscle bellies depending on intraoperative findings and manipulation. There is also an example of performing a longitudinal myotomy with exposure of only the fibrotic band and subsequent release providing relief when esthetics is the only concern (10), and endoscopic approaches to avoid scars (11). The most effective operation noted in the literature is a Ferkel’s procedure (12), involving bipolar release through two incisions with a Z-plasty of the sternal head for lengthening and preservation of normal V-contour of the neck. Subsequent publications have noted that a Z-plasty on the SCM is not required due to similar results with comparable cosmesis (7).

We have also found that the fibrotic band noted on MRI correlated with the area requiring release or resection in this case. In fact, the patient was able to identify the area of most concern, which can be seen in Figure 1. However, the imaging modality of choice for CMT is accepted to be ultrasound, with CMT muscles generally showing up as hyperechogenic bands. A study reviewing infantile torticollis has found that MRI is only positive 30% of the time in patients <1 year of age (13).

In this case, a focused unipolar release was performed as an initial intervention with the option of a completion bipolar release if results were not satisfactory. This decision was driven by patient preference; a more extensive bipolar release was conveyed as the surgeon’s preference given that this was a redo surgery. However, our conclusion, consistent with the results found in the literature, is that bipolar release of both anterior and posterior muscle bellies, with resection if indicated due to extensive adhesions, is the best initial approach to treating neglected CMT in the adult population (14). Additionally, without proper physical therapy short-term results are unlikely to be stable and attention to proper post surgical care should be made. We hope this follow-up study will prove a useful contribution for those patients and providers who may be presented with this rare but difficult problem.

## Ethics Statement

No experimental procedures were being done, therefore consent was not procured for this purpose. Consent for surgery and use of pictures for research was obtained.

## Author Contriibutions

All authors listed, have made substantial, direct, and intellectual contribution to the work, and approved it for publication.

## Conflict of Interest Statement

The authors declare that the research was conducted in the absence of any commercial or financial relationships that could be construed as a potential conflict of interest.
